# More Than Just Anxiety: Math Attitudes as Key Driver of University Choice

**DOI:** 10.1111/nyas.70060

**Published:** 2025-09-20

**Authors:** Maristella Lunardon, Christina Artemenko, Serena Rossi, Hans‐Christoph Nuerk, Krzysztof Cipora

**Affiliations:** ^1^ Department of Psychology University of Tuebingen Tuebingen Germany; ^2^ Neuroscience Area Scuola Internazionale Superiore Di Studi Avanzati (SISSA) Trieste Italy; ^3^ LEAD Graduate School & Research Network University of Tuebingen Tuebingen Germany; ^4^ Centre for Mathematical Cognition Loughborough University Loughborough UK; ^5^ German Center of Mental Health Section Tuebingen Tuebingen Germany

**Keywords:** anxiety, latent profile analysis, math self‐concept, math self‐efficacy, university students

## Abstract

Mathematics anxiety influences not only math performance but also career choices, often leading individuals to avoid math‐intensive fields in higher education. While much research has been devoted to that relation, other factors, such as general and test anxiety, neuroticism, and math‐related attitudes (e.g., math self‐concept and self‐efficacy) have received less attention, although they are related to (or potentially confounded with) math anxiety. In this study, we used latent profile analysis to examine how different profiles of (math) anxiety and attitudes influence students' choice of study programs with varying levels of math content. Our sample consisted of 837 German university students enrolled in programs with low, medium, or high math intensity. We identified seven distinct profiles characterized by different combinations of anxiety and math attitudes. These profiles varied in their distribution across study programs and in the extent to which the presence of mathematics influenced program choice. Notably, differences in study choices were associated much more with math attitudes than with math anxiety. Furthermore, gender distribution varied across profiles, with women being overrepresented in profiles marked by lower math attitudes. These findings underscore the importance of considering math attitudes alongside math anxiety when examining students' academic and career choices.

## Graphical abstract

Mathematics anxiety affects both performance and career choices, yet factors like general anxiety, neuroticism, and math‐related attitudes are less studied. Using latent profile analysis, we identified seven student profiles based on math anxiety and attitudes. These profiles differed in how they were distributed across study programs. Our findings highlight the need to consider math attitudes along with math anxiety when exploring academic pathways and career decision‐making among students.

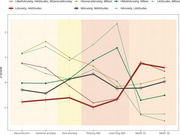

## 1 Introduction

To face the challenges posed by rapid technological and societal changes, there is global demand for experts in the Science, Technology, Engineering and Mathematics (STEM) fields. Therefore, educators and stakeholders aim to attract students in STEM university programs [1, 2], with a particular focus on increasing the participation of girls and women, who are less likely to pursue careers in these fields [3]. The gender imbalance in STEM remains a topic of intense debate, particularly in light of the gender‐equality paradox, that is, the counterintuitive finding that this imbalance is more pronounced in countries with higher gender equality [4]. A crucial step toward both increasing STEM participation and achieving more balanced gender representation is understanding the individual characteristics that make students more likely to pursue careers in STEM. As mathematics is a core knowledge in STEM disciplines, math‐related emotional and attitudinal factors are in the spotlight.

The most frequently investigated and probably most relevant emotional factor in relation to math learning and performance is math anxiety. It is defined as a feeling of tension experienced when handling numbers and solving numerical problems (for a review see Refs. [5, 6]). Its influence extends to students’ career decisions, as math‐anxious individuals tend to avoid pursuing university programs with high math load [7, 8, 9]. Moreover, higher math anxiety is associated with lower grades and reduced enrolment in STEM courses, irrespective of individual math proficiency [10]. Additionally, girls and women tend to report higher math anxiety compared to boys and men [11, 12], a gap even larger in more gender equal countries [13]. However, the specificity of the impact of math anxiety remains a topic of discussion. Math anxiety represents just one of several emotional and motivational factors influencing math‐related choices and outcomes. Therefore, math anxiety warrants further investigation not only in isolation, but also in conjunction with individual differences in more general anxiety types and in math‐specific attitudes.

Previous research has linked math anxiety to different types of anxiety. Meta‐analyses have shown that math anxiety positively correlates with test anxiety, that is, the anxiety experienced in evaluative settings, and with general anxiety, that is, the general tendency to feel anxious about everyday situations, with the former correlation being stronger than the latter [14, 15, 16]. Moreover, recent studies have extended this understanding revealing associations between math anxiety and neuroticism, a personality trait characterized by a disposition toward negative emotions encompassing anxiety, anger, frustration, and feeling of uncontrollability, especially when facing threats [17, 18, 19, 20, 21].

Math anxiety can coexist with other anxiety types in some university students, while others may experience specific academic anxiety such as test and/or math anxiety exclusively. Importantly, individuals characterized by different anxiety combinations, despite sharing similar math anxiety may differ in arithmetic performance [21]. However, it remains unclear whether different combinations of anxiety can influence the choice of a study program, as well as whether and how individuals weigh the presence of mathematics as a determinant in this choice.

Math anxiety is also closely linked with attitudes toward mathematics. In this study, we use the term “math attitudes” to refer to math‐related self‐beliefs (i.e., math self‐concept and math self‐efficacy), distinguishing them from math anxiety, which indicates negative feelings in relation to math and includes a cognitive component represented by worrisome thoughts about math failure [22]. We acknowledge that some frameworks conceptualize both math anxiety and self‐beliefs as dimensions of math attitudes, with the former seen as emotional and the latter as cognitive [23]. However, in our view this distinction between emotional and cognitive dimensions overlooks that math anxiety also entails a cognitive component (i.e., worrisome thoughts) and self‐beliefs also entail an emotional one (e.g., liking the subject).

Math self‐concept refers to an individual's perception of their proficiency in math [24], while math self‐efficacy relates to the confidence in their abilities to solve mathematical tasks [25]. These attitudes are negatively correlated with math anxiety [16, 26] and women tend to have less positive math attitudes than men [27]. Moreover, math self‐concept and self‐efficacy are likely to differ among students in study programs with different math load. For example, higher levels of math self‐efficacy have been associated with increased interest in science or math careers among high school students [28], and higher math self‐concept has been linked to lower dropout rates among first‐year university students in mathematics study programs [29]. However, recent research among university students found no significant association between field of study (STEM vs. non‐STEM) and math self‐efficacy and enjoyment [30]. By considering different types of anxiety and math attitudes simultaneously, we aim to better understand differences between university students in programs with varying math load.

A methodological approach to examine how multiple factors manifest within individuals is the latent profile analysis (LPA), which is used to define subgroups based on patterns of similarities and differences in a set of relevant variables [31]. LPA was previously used to investigate the presence of math anxiety and math attitudes profiles and whether these profiles were associated with arithmetic performance and math‐related behavior in children [32] and high school students [33], revealing different profiles and differences in math performance between profiles. Orbach and Fritz [32] assessed math trait anxiety, math state anxiety, that is, the in‐the‐moment anxiety induced by math‐related stimuli, math self‐concept, and math liking. Children in the profile with high math trait anxiety, low math state anxiety and high math self‐concept and liking showed the highest math performance, while children with high math trait anxiety, low math self‐concept and liking and either average or high math state anxiety showed the lowest math performance. These findings highlight that students with similar levels of trait math anxiety can show substantial differences in math performance depending on other anxiety types and math attitudes. This suggests that math anxiety should not be examined in isolation when explaining mathematical performance. Wang et al. [33] investigated math learning and testing anxiety and math attitudes such as importance, self‐perceived ability, and interest. The best mathematical performance was found in high school students in the profile with modest math testing anxiety and high math attitudes, whereas the lowest math performance was found in high school students in the profiles with average or low math attitudes and high math testing anxiety [33]. Additionally, high school students with both high math testing anxiety and high math attitudes spent more time in math activities after school [33]. Altogether, these findings show that moderate or high levels of math anxiety can coexist with positive math attitudes.

In university students, LPA was used to investigate the presence of anxiety profiles only, including math anxiety, test anxiety, and general anxiety [21]. Five different profiles were identified: (i) with high levels in all anxiety types, (ii) with high levels in all anxiety types except math learning anxiety, (iii) with high levels in math testing and learning anxiety, (iv) with high levels in test anxiety, and (v) with low levels in all anxiety types. Notably, performance in an arithmetic test was equally low in all the profiles with high levels of at least one type of anxiety, compared to the low anxiety profile. Due to math avoidance in students with high anxiety, students with low anxiety may be the majority studying in programs with high math load. This hypothesis was exploratively tested, but the results did not reach statistical significance. It is possible that math avoidance at university is not due to math anxiety only and the combination of different anxiety types with math attitudes could be more informative.

### 1.1 Objectives

Therefore, the first aim of this preregistered exploratory study (https://osf.io/96tsv) is to investigate the specificity of the impact of math anxiety on students’ career choices by considering simultaneously other types of anxiety and math attitudes. We used LPA to examine how different types of anxiety and math attitudes influence university students’ program choice. Based on previous findings, we expected to find profiles with high levels in all anxiety types and low math attitudes, with low anxiety and high math attitudes, and with average anxiety and average math attitudes [21, 32, 33, 34], as well as more diversified profiles, for example, one with high anxiety and high math attitudes [32], and one with high math or test anxiety, low general anxiety and neuroticism, and low math attitudes [21, 34]. However, as this is the first time that profiles including different anxiety types and math attitudes were to be investigated in university students, we could not exclude finding more differentiated profiles than those hypothesized.

Regarding study programs, we expected that low math load study programs may include more students from profiles with high anxiety and low math attitudes [21], while high math load study programs may include more students from profiles with high math attitudes and either high or low anxiety [32, 33]. Additionally, math might not have played a role in the choice for students in the low anxiety and low math attitudes profile. Math might have been avoided particularly by students in profiles with high math anxiety and low math attitudes. Conversely, students in profiles with low math anxiety and high math attitudes may have considered the presence of math in the study program as desirable.

The second aim of this study was to explore gender differences. Rossi et al. [21] observed that women were more likely to belong to the high anxiety profile and the math anxiety profile, whereas men were more prevalent in the low anxiety profile and the test anxiety profile. Similarly, girls were more likely to belong to the profiles with high math anxiety and low math attitudes than boys [32, 33]. Therefore, we expected men to be more represented in profiles with low levels in all anxiety types, except for test anxiety, and high math attitudes [21, 32]. Women were expected to be more represented in profiles with high anxiety and low math attitudes [21, 33]. We also expected gender differences in the profile distribution between math loads and in the association between profiles and math avoidance.

## 2 Materials and Methods

This study used openly accessible data (the AMATUS dataset [18]) and was preregistered in the Open Science Framework (OSF; https://osf.io/96tsv) as a secondary data analysis. In this study, we only included large‐scale online survey data of the university students from the AMATUS (Arithmetic Performance, Mathematics Anxiety and Attitudes in primary school Teachers and University Students) dataset. A detailed description of the dataset, codebook and measures can be retrieved from OSF (https://osf.io/gszpb/). The code used for the main analyses reported here can be retrieved from OSF (https://osf.io/z5egb/).

### 2.1 Participants

Participants were students at the University of Tuebingen, recruited via university e‐mails. The online survey was completed by 1049 students. Participants were removed from the dataset if they were not native German speaker (*n* = 34), did not complete the survey (*n* = 110), responded dishonestly (*n* = 4), reported a very or extremely noisy environment during study completion (*n* = 36), or took an extremely long time to complete the survey (i.e., more than 30 min, *n* = 17). Additionally, we removed participants who entered a study program name that could not be traced back to any existing degree course (*n* = 2), so they could not be assigned to a math load category. After removing multivariate outliers during data analysis (*n* = 9), the final sample included 837 participants (246 men and 591 women, age: *M* = 23.57; SD = 4.19): 379 were in a study program with a low math load (85 men and 294 women); 341 were in a study program with a medium math load (98 men and 243 women); 117 were in a study program with a high math load (63 men and 54 women). This sample is sufficient to run LPA, as a minimum of 300–500 observations is usually recommended [35, 36]. As compensation, participants were offered to enter a raffle for vouchers. Ethical approval was obtained by the Ethics Committee for Psychological Research of the University of Tuebingen.

### 2.2 Materials

#### 2.2.1 Neuroticism

Neuroticism was assessed by the eight respective items of the German short version of the Big Five Inventory (BFI‐K) [37]. Each item corresponded to a statement and participants had to rate their agreement on a 5‐point Likert scale (1 = very incorrect, 5 = very correct). The neuroticism score is the sum of each item response, with higher values corresponding to higher neuroticism (*Theoretical Range* = 8–40). In the current sample, ordinal alpha was 0.88.

#### 2.2.2 General Anxiety

General trait anxiety was assessed with the German version of the Generalized Anxiety Disorder Screener (GAD‐7) [38]. Participants were asked to indicate how often they had experienced the emotional states described in each of the seven items during the last 2 weeks (1 = not at all, 2 = several days, 3 = more than half the days, 4 = nearly every day). The general trait anxiety score is the sum of each item response, with higher values corresponding to higher anxiety levels (*Theoretical Range* = 7–28). In the current sample, ordinal alpha was 0.86.

#### 2.2.3 Test Anxiety

Test anxiety was assessed by the original English short version of the Test Anxiety Inventory (TAI‐short) [39], translated into German. Participants were asked to indicate on a 4‐point Likert scale how much they agreed on five items describing states during examinations (1 = not at all, 4 = very). The test anxiety score is the sum of each item response, with higher values corresponding to higher anxiety levels (*Theoretical Range* = 5–20). In the current sample, ordinal alpha was 0.87.

#### 2.2.4 Math Anxiety

Math anxiety was assessed by the Abbreviated Math Anxiety Scale (AMAS) [40]. The German translation by Dietrich et al. [41] was slightly modified to whole sentences to better adapt the German version to the original English one. Participants were asked to indicate how anxious they would feel in each math‐related situation described in the nine items using a 5‐point Likert scale (1 = little anxious, 5 = very anxious). The AMAS has two subscales: learning math anxiety and math testing anxiety. Total scores, as the sum of single item responses, were calculated for each subscale with higher values corresponding to higher anxiety levels (learning math anxiety: *Theoretical Range* = 5–25; math testing anxiety: *Theoretical Range* = 4–20). In the current sample, ordinal alpha was 0.90 for both the math learning and the math testing anxiety subscales.

#### 2.2.5 Math Self‐Concept

Math self‐concept was assessed by the mathematical ability subscale of the German adaptation [42] of the Self‐Description Questionnaire III (SDQ‐III) [43]. The scale consisted of four statements regarding ability in math. Participants were asked to indicate on a 4‐point Likert scale how much they agreed with each statement (1 = do not agree at all, 4 = absolutely agree). The math self‐concept score is the sum of each item responses with higher values corresponding to higher self‐concept (*Theoretical Range* = 4–16). In the current sample, ordinal alpha was 0.93.

#### 2.2.6 Math Self‐Efficacy

Math self‐efficacy was assessed by German items from the PISA 2012 study [44]. For six items, participants were asked to indicate on a 4‐point Likert scale how confident they feel in solving math tasks (1 = not at all confident, 4 = very confident). The math self‐efficacy score is the sum of the item responses with higher values corresponding to higher self‐efficacy (*Theoretical Range* = 6–24). In the current sample, ordinal alpha was 0.89.

#### 2.2.7 Math Load

Participants had to indicate the math load in their study program in one of three categories: low, medium, or high. The study programs were categorized based on our expertise and on the study program handbook of the university. For each response option, examples were provided: for example, computer science, physics, and mathematics for the “high math load”; for example, chemistry, psychology, and economics for the “medium math load”; for example, literature, history, and pedagogy for “low math load.” We extended the typical STEM/non‐STEM classifications, as we assumed that math load in some STEM programs (e.g., mathematics or physics) is higher than in other STEM programs (e.g., biology or medicine), and as some non‐STEM programs include mathematics or statistic teaching (e.g., psychology or sociology). To double‐check participant selection, they additionally had to name their study program (for anonymity these responses were not reported in the openly shared dataset) and the researchers verified the pertinence of the category. If participants studied in more than one study program, they were asked to choose the appropriate category for their major study program and, in case of two major study programs, for their study program including a higher amount of math. If their study program was not included in any of these categories, they could choose a fourth option (“other”) and indicate their study program; in this case, the study program was assigned to a math load category by the researchers after consulting the study program handbook. See Table S1 (available at https://osf.io/3ehqc) for the list of university programs included in each math load category.

#### 2.2.8 Influence of Math Load on the Choice of the Study Program

Participants had to indicate how much and in what manner the math load in the study program influenced their study program choice on a 9‐point Likert scale. Lower values indicate that the math load mattered in the sense that led them to avoid math courses (“For the choice of my study program the math load played a role because I wanted to avoid math subjects.”). Higher values indicate that the study program was chosen because of the willingness to pursue math courses (“For the choice of my study program the math load played a role because I wanted to take math subjects.”). Medium values indicate that math load did not play any role in the choice of the study program (“For the choice of my study program the math load played no role.”).

#### 2.2.9 Subject Liking

Liking in math and humanities was assessed by a single item each. Participants were asked to indicate on a 5‐point Likert scale how much they agree with the statements “I like math/humanities.” (1 = do not agree at all, 5 = absolutely agree). Higher scores correspond to higher levels of liking.

#### 2.2.10 Arithmetic Performance

Arithmetic performance was assessed by an arithmetic speed test. Participants had to solve as many as possible of 40 arithmetic problems within 2 min [21, 45]. The test was designed similar to the Math4Speed [46] but mixed all basic arithmetic operations (addition, subtraction, multiplication, and division) of varying difficulty (one‐ to three‐digit‐numbers, with/without carrying/borrowing). The arithmetic problems were presented in a fixed random order (constant for all participants). Participants were instructed to solve the problems in the presented order, mentally, and without using a calculator. The score for arithmetic performance was operationalized as the number of correctly solved problems. The final score was retained only for participants who did not skip any item (*n* = 729).

#### 2.2.11 Math Achievement

As a measure of math achievement, we used the last school mathematics grade reported by participants, expressed in the German grading system, as numbers from 1 to 6, with 1 being the best grade.

### 2.3 Procedure

The study was implemented in German in the SoSci Survey platform [47]. All participants gave informed consent via mouse click before starting the survey. First, participants were asked for basic demographic information: age (in years), gender (male or female), native language (German or other), and last school mathematics grade. Then, they were asked about the math load of their study program and the influence it had on their study program choice. Afterward, they completed the questionnaires on neuroticism, general anxiety, test anxiety, math anxiety, math self‐concept, and math self‐efficacy. Then, participants responded to items designed for the purpose of the study about how much they liked math and humanities. Subsequently, they performed the arithmetic speed task and finally responded to some quality‐check questions for web‐based research asking whether they responded honestly and how noisy their environment was during survey completion. The survey also included other questionnaires, which were not part of the present study [18]. The survey was designed to last about 15 min, but no time constraints were forced, except for the arithmetic task, so each participant completed the items at their own pace.

### 2.4 Data Analysis

We report both frequentist and Bayesian statistical analyses according to our preregistration. First, we identified multivariate outliers computing the Mahalanobis distance with *p* < 0.001 and removed them (*n* = 9). Then, we computed descriptive statistics and zero‐order correlations on the whole sample and separately for each math load sample (i.e., low, medium, high).

Afterward, we conducted the LPA. The analysis plan was preregistered, and a detailed description of statistical tests and procedures is available at https://osf.io/96tsv. We estimated models considering math learning and testing anxiety, general anxiety, test anxiety, neuroticism, math self‐concept, and math self‐efficacy as dependent variables. The choice of the model was based on the statistical fit, assessed with Bayesian information criterion (BIC), Akaike information criterion (AIC), bootstrap likelihood ratio test (BLRT), entropy, and posterior probabilities. We also considered the parsimony in the number of profiles and the theoretical significance of the identified profiles as indicators [31, 48].

As a validity check for the profiles, we investigated profile differences in liking of math, liking of humanities, and arithmetic performance. For analyses with arithmetic performance, participants who were not solving the arithmetic problems in order (skippers) were excluded (*n* = 108). We expected to find higher levels of math liking and higher arithmetic performance in profiles with low (math) anxiety and higher math self‐concept and self‐efficacy, and higher levels of humanities liking and lower arithmetic performance in profiles with high math anxiety and low math self‐concept and self‐efficacy. To do so, we ran three one‐way ANOVAs with profile as a factor. Additionally, we conducted a *χ^2^* test to investigate whether the number of skippers in the arithmetic task was different in the profiles, with the expectation that skippers would be more likely to display a profile characterized by negative math emotions and attitudes.

For the main analyses, we conducted the following profile comparisons: we investigated whether the profiles differed in the proportion of students from low, medium and high math load by calculating *χ^2^* tests; we compared the profiles on the influence that math load had in the study program choice with a one‐way ANOVA and a significant main effect was further examined by Bonferroni–Holm‐corrected post hoc tests. Finally, we investigated gender differences: we calculated *χ^2^* tests comparing the proportion of women and men between the profiles; we compared the proportion of math load in each profile for men and women with the Cochran–Mantel–Haenszel test; we investigated gender differences in the association between profiles and the influence that math load had in the study program choice with an ANOVA with gender and profiles as between‐subject factors and a significant interaction was further examined by Bonferroni–Holm‐corrected post hoc tests.

Data were analyzed with the statistical software R version 4.2.2 [49], and for the LPA we used the “*TidyLPA*” package [50]. The R code for the analyses can be found at https://osf.io/z5egb/files/osfstorage.

## 3 Results

Table 1 reports the descriptive statistics for the study variables on the entire sample and separately for math load category. Frequentist and Bayesian correlations are reported in Table 2 for the entire sample and in Tables S2–S4 (https://osf.io/3ehqc) for the three math load categories. In line with the previous literature, math anxiety correlated positively with other forms of anxiety and negatively with math self‐concept and math self‐efficacy. Similarly, better arithmetic performance was associated with lower (math) anxiety and higher math self‐concept and math self‐efficacy.

**TABLE 1:** Descriptive statistics in the entire sample, including skewness, kurtosis, and their standard errors (*SE*), and separately by math load.

	Whole sample			Low math load	Medium math load	High math load
	*n* = 837			*n* = 379	*n* = 341	*n* = 117
	*M* (*SD*)	Skewness (*SE*)	Kurtosis (*SE*)	*M* (*SD*)	*M* (*SD*)	*M* (*SD*)
Age	23.57 (4.19)	2.33 (0.08)	11.05 (0.17)	23.69 (4.20)	23.32 (4.09)	23.91 (4.46)
Neuroticism	23.61 (6.11)	0.16 (0.08)	−0.64 (0.17)	24.26 (6.15)	23.25 (6.09)	22.56 (5.80)
General anxiety	12.58 (3.95)	1.12 (0.08)	1.00 (0.17)	13.09 (4.12)	12.15 (3.86)	12.20 (3.50)
Test anxiety	11.29 (3.82)	0.39 (0.08)	−0.68 (0.17)	11.44 (3.81)	11.26 (3.77)	10.93 (4.00)
Math anxiety total	18.55 (6.44)	0.61 (0.08)	−0.23 (0.17)	20.44 (6.68)	17.54 (6.03)	15.38 (4.71)
Math learning anxiety	7.31 (3.03)	1.48 (0.08)	1.75 (0.17)	8.11 (3.40)	6.92 (2.70)	5.88 (1.68)
Math testing anxiety	11.24 (4.06)	0.08 (0.08)	−0.92 (0.17)	12.33 (3.92)	10.62 (4.02)	9.50 (3.67)
Math self‐concept	11.44 (3.12)	−0.33 (0.08)	−0.81 (0.17)	9.98 (3.08)	12.35 (2.68)	13.50 (2.18)
Math self‐efficacy	20.35 (3.35)	−0.84 (0.08)	0.06 (0.17)	18.80 (3.51)	21.30 (2.70)	22.59 (1.97)
Influence of math load on study choice	5.05 (1.96)	−0.03 (0.08)	−0.07 (0.17)	3.98 (1.64)	5.50 (1.56)	7.23 (1.64)
Math liking	3.18 (1.31)	−0.17 (0.08)	−1.06 (0.17)	2.57 (1.27)	3.51 (1.11)	4.21 (0.89)
Humanities liking	3.88 (1.10)	−0.80 (0.08)	−0.16 (0.17)	4.53 (0.70)	3.42 (1.09)	3.16 (1.07)
Arithmetic performance^a^	13.67 (6.68)	0.70 (0.09)	0.73 (0.18)	12.19 (6.44)	14.38 (6.21)	16.02 (7.63)

^a^After removing subjects that skipped items: *n* = 729 in the entire sample, *n* = 319 in low math load, *n* = 302 in medium math load, *n* = 108 in high math load.

**TABLE 2:** Correlations between the study variables, with 95% confidence intervals (*CI*) and Bayes factors (*BF*_*10*_).

		1	2	3	4	5	6	7	8	9	10	11
1. Neuroticism		—										
2. General anxiety	*r* *CI* *BF*_*10*_	0.61*** [0.56; 0.65] >100	—									
3. Test anxiety	*r* *CI* *BF*_*10*_	0.37*** [0.31; 0.43] >100	0.33*** [0.27; 0.39] >100	—								
4. Math anxiety total	*r* *CI* *BF*_*10*_	0.33*** [0.27; 0.39] >100	0.35*** [0.29; 0.40] >100	0.39*** [0.33; 0.45] >100	—							
5. Math learning anxiety	*r* *CI* *BF*_*10*_	0.24*** [0.17; 0.30] >100	0.30*** [0.24; 0.36] >100	0.25*** [0.18; 0.31] >100	0.88*** [0.86; 0.89] >100	—						
6. Math testing anxiety	*r* *CI* *BF*_*10*_	0.35*** [0.29; 0.41] >100	0.32*** [0.26; 0.38] >100	0.44*** [0.38; 0.49] >10000	0.93*** [0.92; 0.94] NA	0.64*** [0.60; 0.68] >100	—					
7. Math self‐concept	*r* *CI* *BF*_*10*_	−0.19*** [−0.25; −0.12] >100	−0.19*** [−0.25; −0.12] >100	−0.19*** [−0.26; −0.13] >100	−0.64*** [−0.68; −0.60] >100	−0.55*** [−0.59; −0.50] >100	−0.61*** [−0.65; −0.56] >100	—				
8. Math self‐efficacy	*r* *CI* *BF*_*10*_	−0.25*** [−0.31; −0.18] >100	−0.17*** [−0.23; −0.10] >100	−0.16*** [−0.23; −0.10] >100	−0.50*** [−0.55; −0.45] >100	−0.45*** [−0.50; −0.39] >100	−0.46*** [−0.51; −0.40] >100	0.55*** [0.50; 0.59] >100	—			
9. Influence of math load on study choice	*r* *CI* *BF*_*10*_	−0.12*** [−0.19; −0.05] 34.66	−0.09** [−0.16; −0.03] 3.46	−0.10** [−0.16; −0.03] 4.08	−0.44*** [−0.49; −0.38] >100	−0.36*** [−0.41; −0.29] >100	−0.43*** [−0.48; −0.37] >100	0.62*** [0.58; 0.66] >100	0.41*** [0.35; 0.46] >100	—		
10. Math liking	*r* *CI* *BF*_*10*_	−0.16*** [−0.23; −0.10] >100	−0.13*** [−0.19; −0.06] 76.49	−0.14*** [−0.21; −0.08] >100	−0.58*** [−0.62; −0.53] >100	−0.46*** [−0.51; −0.40] >100	−0.58*** [−0.62; −0.53] >100	0.80*** [0.77; 0.82] >100	0.51*** [0.46; 0.56] >100	0.65*** [0.61; 0.69] >100	—	
11. Humanities liking	*r* *CI* *BF*_*10*_	0.04 [−0.02; 0.11] 0.17	0.06 [−0.10; 0.13] 0.41	−0.03 [−0.10; 0.04] 0.12	0.22*** [0.16; 0.29] >100	0.22*** [0.15; 0.28] >100	0.19*** [0.12; 0.25] >100	−0.30*** [−0.36; −0.24] >100	−0.28*** [−0.34; −0.22] >100	−0.32*** [−0.38; −0.25] >100	−0.27*** [−0.33; −0.21] >100	—
12. Arithmetic performance	*r* *CI* *BF*_*10*_	−0.12*** [−0.20; −0.05] 24.13	−0.07 [−0.14; 00] 0.55	−0.07 [−0.14; 0.01] 0.41	−0.25*** [−0.32; −0.18] >100	−0.19*** [−0.26; −0.12] >100	−0.25*** [−0.32; −0.18] >100	0.31*** [0.24; 0.38] >100	0.33*** [0.27; 0.40] > 100	0.23*** [0.16; 0.30] >100	0.31*** [0.24; 0.37] >100	−0.14*** [−0.21; −0.06] 79.65

*p < 0.05; **p < 0.01; ***p < 0.001.

### 3.1 Latent Profile Analysis

Before running LPA, we conducted Mardia's test and Energy test for multivariate normality, both of which yielded statistically significant results (*p* < 0.001). Normality tests are generally sensitive [51]. Based on visual inspection of the distributions and the examination of skewness and kurtosis, we believe that any deviations from normality are minimal and unlikely to substantially impact the LPA results. Therefore, we conducted the LPA using the maximum likelihood estimator, instead of maximum likelihood with robust standard error, and thus deviated from the study preregistration. Additionally, to ensure the results were robust across varying variance and covariance structures, we computed the cross‐validation error for each model.

**FIGURE 1:**
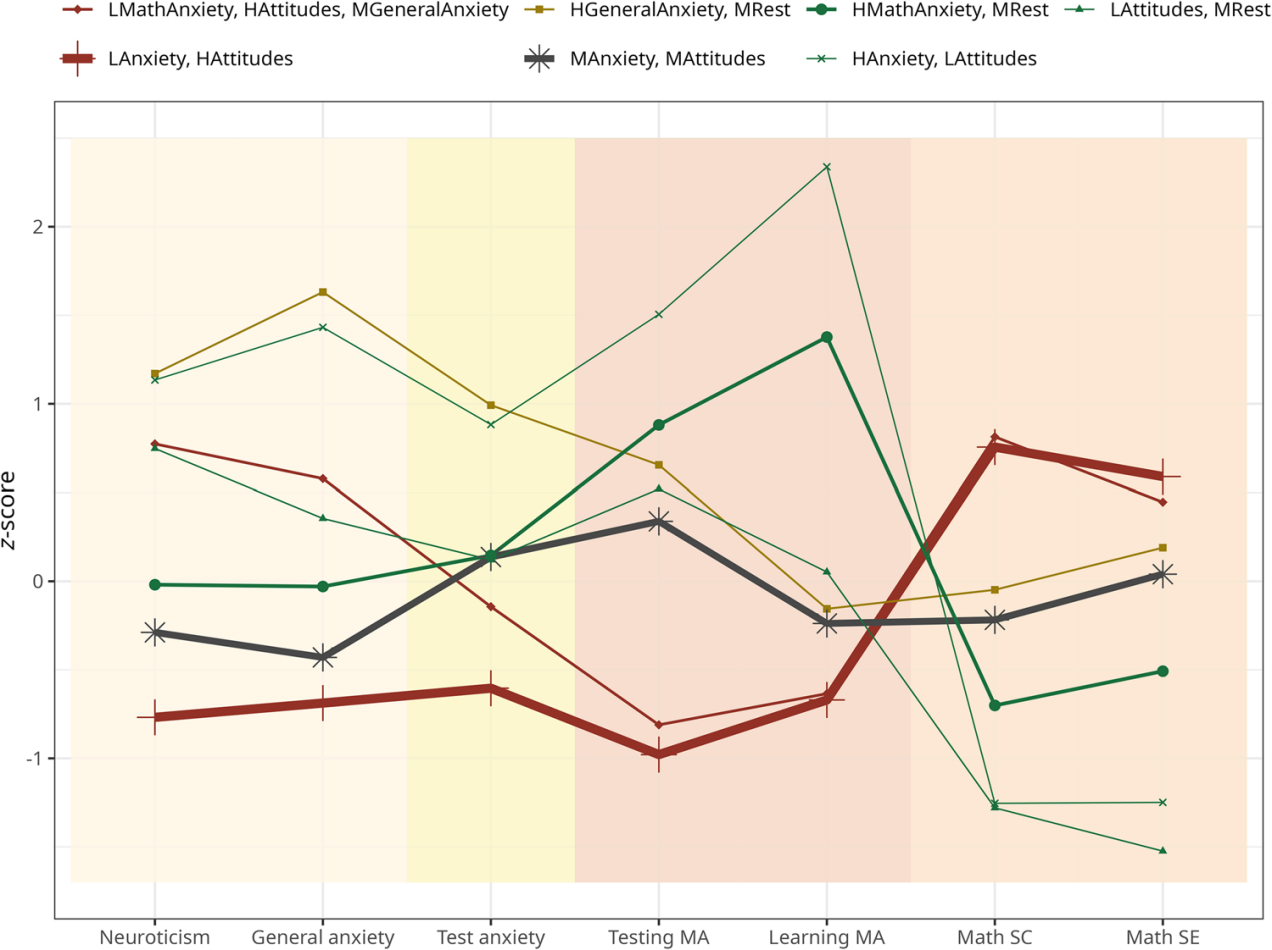
Characteristics of each profile. *Z*‐scores of each profile in each latent profile analysis variable. The line thickness is proportional to profile size. Green profiles are characterized by negative emotions and attitudes toward math. Red profiles are characterized by positive attitudes toward math. The *Medium anxiety and attitudes* is in gray and the *High general anxiety, medium rest* is in yellow. *Profiles*: LMathAnxiety, HAttitudes, MGeneralAnxiety = Low math anxiety, high attitudes, medium general anxiety; LAnxiety, HAttitudes = Low anxiety, high attitudes; HGeneralAnxiety, MRest = High general anxiety, medium rest; MAnxiety, MAttitudes = Medium anxiety and attitudes; HMathAnxiety, MRest = High math anxiety, medium rest; HAnxiety, LAttitudes = High anxiety, low attitudes; LAttitudes, MRest = Low attitudes, medium rest. MA, math anxiety; SC, self‐concept; SE, self‐efficacy.

We tested 10 models, either with equal variance and covariance set to 0 (EEI) or with equal variance and equal covariance (EEE), in which we added iteratively one profile at the time. Their fit information is reported in Table 3. The cross‐validation errors were close to zero. EEI models had in general higher entropy than EEE models, suggesting better classification. The BLRT *p* values < 0.05 indicate significant differences between models with different numbers of profiles (e.g., model with *n* + 1 profile better than model with *n* profiles) in the EEI models. According to our preregistered criteria, we discarded models with nine and ten profiles because the smallest group included less than 5% of the entire sample. The model with eight profiles displayed the lowest AIC and BIC. The visual inspection of the resulting profiles in the seven‐profile and eight‐profile solutions led us to opt for the more parsimonious solution, that is, the model with seven profiles (Figure 1).

**TABLE 3:** Model fit indicators for latent profile solutions.

Number of profiles	AIC	BIC	Entropy	*N*_min_	BLRT *p* value	Cross‐validation error (SE)
EEI						
1	16648	16714	1	1		
2	15374	15478	0.82	0.42	0.010	0.038 (0.006)
3	14881	15022	0.85	0.15	0.010	0.059 (0.005)
4	14729	14909	0.84	0.08	0.010	0.073 (0.009)
5	14661	14879	0.79	0.08	0.010	0.078 (0.009)
6	14588	14843	0.79	0.07	0.010	0.088 (0.008)
**7**	**14388**	**14681**	**0.81**	**0.06**	**0.010**	**0.103 (0.012)**
8	14323	14655	0.81	0.05	0.010	0.080 (0.013)
9	14280	14648	0.80	0.04	0.010	0.136 (0.011)
10	14258	14665	0.79	0.03	0.010	0.121 (0.012)
EEE						
1	14653	14819	1	1		
2	14604	14807	0.66	0.48	0.010	0.056 (0.007)
3	14281	14523	0.74	0.20	0.010	0.073 (0.01)
4	14181	14460	0.77	0.08	0.010	0.081 (0.009)
5	14138	14455	0.78	0.07	0.010	0.082 (0.01)
6	14130	14485	0.76	0.05	0.059	0.082 (0.012)
7	14146	14538	0.69	0.00	0.960	0.080 (0.009)
8	14131	14561	0.70	0.06	0.050	0.128 (0.011)
9	14002	14470	0.74	0.05	0.010	0.152 (0.015)
10	14015	14522	0.73	0.02	0.990	0.153 (0.015)

*Note*: Entropy ranges from 0 to 1, with higher values indicating better classification. **Bold** indicates the number of profiles selected. Abbreviations: AIC, Akaike information criterion; BIC, Bayesian information criterion; BLRT *p* value, *p* value of the bootstrapped likelihood ratio test; EEE, models with equal variance and equal covariance; EEI, models with equal variance and covariance set to 0; *N*_min_, proportion of participants in the smallest profile; SE, standard error of the cross‐validation error.

The selected model includes three profiles characterized by negative emotions and attitudes toward mathematics:

- Profile 1*. Low attitudes, medium rest (LAttitudes, MRest)*: low math self‐concept and self‐efficacy, average levels in all the other variables.
- Profile 2*. High anxiety, low attitudes (HAnxiety, LAttitudes)*: high levels in all anxiety types, low math self‐concept and self‐efficacy.
- Profile 3. *High math anxiety, medium rest (HMathAnxiety, MRest)*: high testing and learning math anxiety, average levels in all the other variables.

Two profiles are characterized by medium emotions and attitudes toward mathematics:

- Profile 4*. Medium anxiety and attitudes (MAnxiety, MAttitudes)*: average levels in all the variables.
- Profile 5. *High general anxiety, medium rest (HGeneralAnxiety, MRest)*: high neuroticism, general and test anxiety, and average math anxiety and attitudes.

Two profiles are characterized by positive emotions and attitudes toward mathematics:

- Profile 6*. Low anxiety, high attitudes (LAnxiety, HAttitudes)*: low levels in all anxiety types and high math self‐concept and self‐efficacy.
- Profile 7*. Low math anxiety, high attitudes, medium general anxiety (LMathAnxiety, HAttitudes, MGeneralAnxiety)*: low math anxiety, high math self‐concept and self‐efficacy, average neuroticism, general and test anxiety.

Descriptive statistics for the seven profiles are reported in Table 4.

**TABLE 4:** Descriptive statistics of the study variables in the seven profiles; mean (SE).

	Low attitudes, medium rest	High anxiety, low attitudes	High math anxiety, medium rest	Medium anxiety, attitudes	High general anxiety, medium rest	Low anxiety, high attitudes	Low math anxiety, high attitudes, medium general anxiety
*n*	57	54	114	196	70	256	90
Neuroticism	28.18 (4.47)	30.54 (4.45)	23.49 (5.25)	21.85 (4.39)	30.76 (4.29)	18.92 (4.05)	28.33 (3.45)
General anxiety	13.98 (2.64)	18.24 (3.95)	12.46 (2.66)	10.88 (2.04)	19.03 (3.72)	9.86 (1.74)	14.87 (3.05)
Test anxiety	11.75 (3.42)	14.67 (3.56)	11.85 (3.51)	11.82 (3.53)	15.09 (3.73)	8.98 (2.88)	10.74 (3.13)
Math learning anxiety	13.35 (2.9)	17.35 (1.88)	14.82 (2.34)	12.61 (2.21)	13.90 (2.59)	7.26 (2.06)	7.94 (2.12)
Math testing anxiety	7.47 (1.64)	14.41 (2.44)	11.49 (1.55)	6.59 (1.38)	6.84 (1.71)	5.28 (0.66)	5.39 (0.79)
Math self‐concept	7.44 (2.04)	7.52 (2.29)	9.25 (2.23)	10.76 (2.29)	11.29 (2.35)	13.80 (1.99)	13.98 (1.80)
Math self‐efficacy	15.25 (2.42)	16.17 (3.92)	18.65 (2.99)	20.48 (2.72)	20.99 (2.12)	22.32 (1.95)	21.84 (2.41)
Math liking	1.68 (0.83)	1.81 (0.99)	2.40 (1.09)	2.97 (1.08)	3.04 (1.06)	4 00 (0.99)	4.16 (0.95)
Humanities liking	4.30 (0.98)	4.46 (0.75)	4.28 (0.77)	3.87 (1.08)	3.60 (1.38)	3.61 (1.14)	3.80 (1.08)
Arithmetic performance	9.59 (5.41)	10.16 (5.73)	12.51 (6.47)	13.21 (6.50)	12.93 (5.73)	15.43 (6.61)	15.13 (7.13)
Influence of math load on study choice	3.56 (1.75)	3.52 (1.81)	3.99 (1.72)	4.79 (1.88)	4.93 (1.55)	6.03 (1.63)	6.14 (1.67)

### 3.2 Profile Validity

**FIGURE 2:**
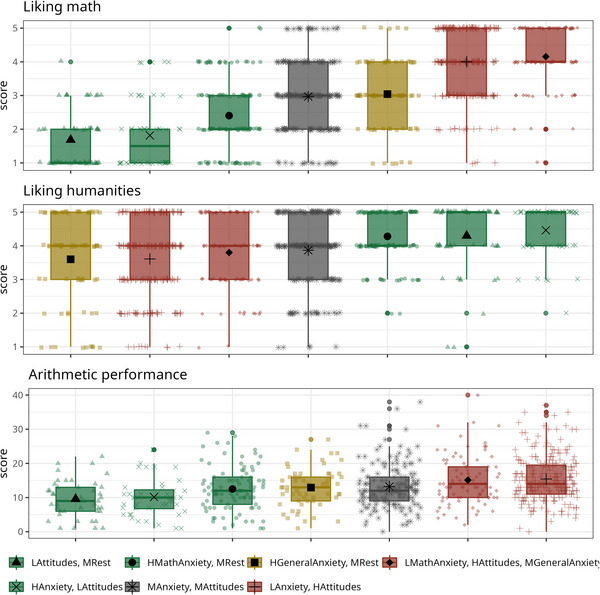
Comparisons between profiles in the criterion validity variables. Transparent points represent individual scores. Profiles were ordered for each variable separately. *Profiles*: LMathAnxiety, HAttitudes, MGeneralAnxiety = Low math anxiety, high attitudes, medium general anxiety; LAnxiety, HAttitudes = Low anxiety, high attitudes; HGeneralAnxiety, MRest = High general anxiety, medium rest; MAnxiety, MAttitudes = Medium anxiety and attitudes; HMathAnxiety, MRest = High math anxiety, medium rest; HAnxiety, LAttitudes = High anxiety, low attitudes; LAttitudes, MRest = Low attitudes, medium rest.

**FIGURE 3:**
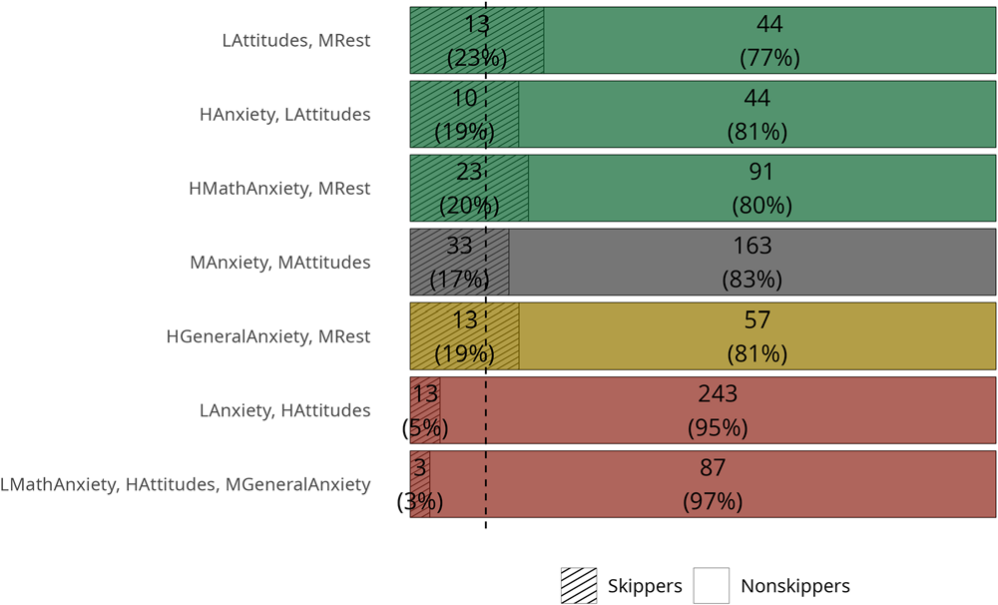
Proportion of skippers and nonskippers in the arithmetic task in each profile. The vertical dashed line represents the percentage of skippers in the entire sample (13%). Profiles: LMathAnxiety, HAttitudes, MGeneralAnxiety = Low math anxiety, high attitudes, medium general anxiety; LAnxiety, HAttitudes = Low anxiety, high attitudes; HGeneralAnxiety, MRest = High general anxiety, medium rest; MAnxiety, MAttitudes = Medium anxiety and attitudes; HMathAnxiety, MRest = High math anxiety, medium rest; HAnxiety, LAttitudes = High anxiety, low attitudes; LAttitudes, MRest = Low attitudes, medium rest.

We first checked the profile validity. The ANOVA results indicated a significant effect of profile for math liking (*F*(6, 830) = 90.94, *p* < 0.001, *BF*_*10*_ > 100), humanities liking (*F*(6, 830) = 10.50, *p* < 0.001, *BF*_*10*_ > 100) and arithmetic performance (*F*(6, 722) = 9.60, *p* < 0.001, *BF*_*10*_ > 100). Post hoc comparisons are reported in Table S5 (https://osf.io/3ehqc). As expected, students in the *Low attitudes*, *medium rest*, *High anxiety*, *low attitudes*, and the *High math anxiety*, *medium rest* profiles reported lower math liking compared to all the other profiles, and showed lower arithmetic performance compared to the *Low anxiety*, *high attitudes* and *Low math anxiety*, *high attitudes*, *medium general anxiety* profiles. The *High general anxiety*, *medium rest*, *Low anxiety*, *high attitudes*, and the *Low math anxiety*, *high attitudes*, *medium general anxiety* profiles reported greater humanities liking compared to the other profiles (Figure 2). Moreover, the number of skippers was different across profiles (*χ* ^2^(6) = 37.84, *p* < 0.001, Cramér's *V* = 0.21): participants with positive attitudes toward mathematics are the least likely to skip items in the arithmetic task compared to other profiles (Figure 3). Overall, these results point to the validity of the profiles.

### 3.3 Profile Comparison

**FIGURE 4:**
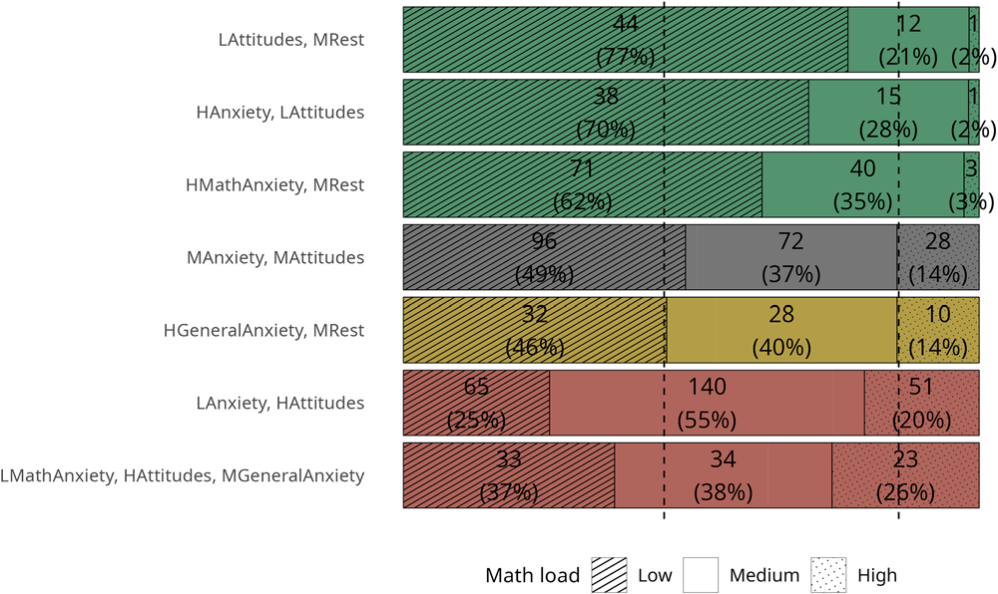
Proportion of students enrolled in low, medium, or high math load study programs in each profile. The vertical dashed lines represent the percentages of students in the three math load groups in the entire sample: 45% in the low math load group, 41% in the medium math load group, 14% in the high math load group. *Profiles*: LMathAnxiety, HAttitudes, MGeneralAnxiety = Low math anxiety, high attitudes, medium general anxiety; LAnxiety, HAttitudes = Low anxiety, high attitudes; HGeneralAnxiety, MRest = High general anxiety, medium rest; MAnxiety, MAttitudes = Medium anxiety and attitudes; HMathAnxiety, MRest = High math anxiety, medium rest; HAnxiety, LAttitudes = High anxiety, low attitudes; LAttitudes, MRest = Low attitudes, medium rest.

After verifying the profile validity, the profiles were compared in the variables of interest. The proportion of students from low, medium, and high math load differed between profiles (*χ* ^2^(12) = 111.17, *p* < 0.001, Cramér's *V* = 0.26). We conducted post hoc analyses using standardized residuals [52] with a Bonferroni‐corrected alpha level of 0.0024 (0.05/21). The students displaying the *Low math anxiety*, *high attitudes*, *medium general anxiety* profiles were significantly more likely to be enrolled in a program with high math load than expected. Also, students with the *Low anxiety*, *high attitudes* profile were more likely to be in a program with high or medium load and less likely to be in a program with low math load, while the contrary was found for the *High math anxiety*, *medium rest* and *Low attitudes*, *medium rest* profiles. Students displaying the *High anxiety*, *low attitudes* profile were more likely to be in a program with low math content (Figure 4).

**FIGURE 5:**
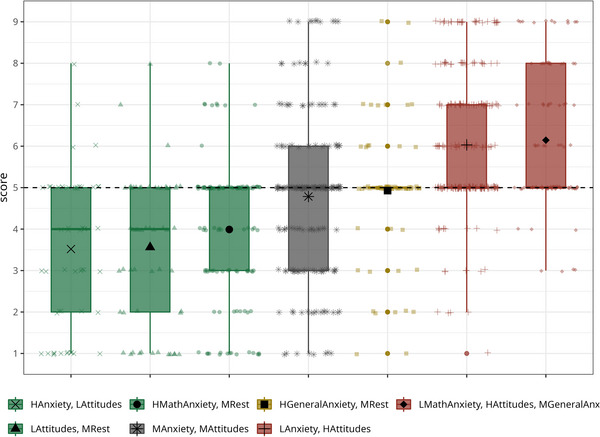
Comparisons between profiles in math load influence on study choice. Lower values indicate that the math load mattered in the sense that led students to avoid math courses. Higher values indicate that the study program was chosen because of the willingness to pursue math courses. Medium values (dashed line) indicate that math load did not play any role in the choice of the study program. Transparent points represent individual scores. Profiles are in ascending order. *Profiles*: LMathAnxiety, HAttitudes, MGeneralAnxiety = Low math anxiety, high attitudes, medium general anxiety; LAnxiety, HAttitudes = Low anxiety, high attitudes; HGeneralAnxiety, MRest = High general anxiety, medium rest; MAnxiety, MAttitudes = Medium anxiety and attitudes; HMathAnxiety, MRest = High math anxiety, medium rest; HAnxiety, LAttitudes = High anxiety, low attitudes; LAttitudes, MRest = Low attitudes, medium rest.

The ANOVA with the influence that math load had in the study program choice as a dependent variable and profile as a factor yielded a significant result (*F*(6, 830) = 42.14, *p* < 0.001, *BF*_*10*_ > 100; see Table S5 for post hoc results, https://osf.io/3ehqc). Students displaying the *Low attitudes*, *medium rest*, *High anxiety*, *low attitudes*, and the *High math anxiety*, *medium rest* profiles more consistently reported to have chosen their study programs to avoid mathematics compared to all the other profiles (*p* < 0.002). Students in the *Low anxiety*, *high attitudes* and the *Low math anxiety*, *high attitudes*, *medium general anxiety* profiles also had a significantly different score from the profiles characterized by average emotions (*p* < 0.001) and reported to have chosen their study program to study mathematics (Figure 5).

### 3.4 Gender Differences

**FIGURE 6:**
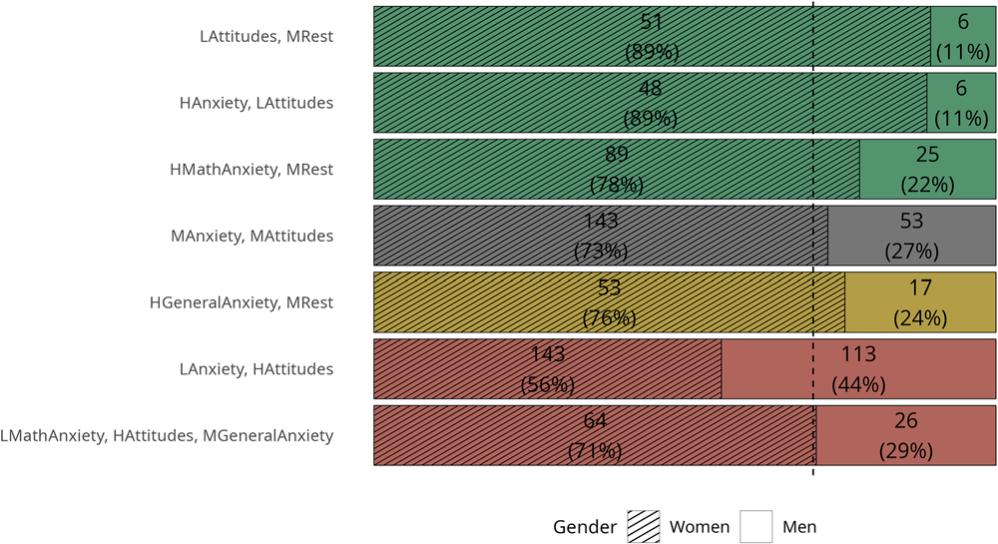
Proportion of women and men in each profile. The vertical dashed line represents the percentage of women in the entire sample (71%). *Profiles*: LMathAnxiety, HAttitudes, MGeneralAnxiety = Low math anxiety, high attitudes, medium general anxiety; LAnxiety, HAttitudes = Low anxiety, high attitudes; HGeneralAnxiety, MRest = High general anxiety, medium rest; MAnxiety, MAttitudes = Medium anxiety and attitudes; HMathAnxiety, MRest = High math anxiety, medium rest; HAnxiety, LAttitudes = High anxiety, low attitudes; LAttitudes, MRest = Low attitudes, medium rest.

The proportion of men and women differed between profiles (*χ* ^2^(6) = 49.78, *p* < 0.001, Cramér's *V* = 0.24). Post hoc analysis using standardized residuals, with an alpha level of 0.0036 (0.05/14) showed that women were significantly more likely to display the profiles with *High anxiety*, *low attitudes* and *Low attitudes*, *medium rest*, and less likely to display the *Low anxiety*, *high attitudes* profile. The opposite pattern was found for men (Figure 6).

We conducted exploratory analyses to investigate joint effects of profile membership and gender. We compared the proportion of students in the three math load categories in each profile in men and in women with the Cochran–Mantel–Haenszel test, and the difference was statistically significant (*χ^2^*_MH_ = 99.66, df = 12, *p* < 0.001). Post hoc analysis on standardized residuals for the association between profiles and math load within gender (Bonferroni‐corrected alpha level = 0.05/42 = 0.0012) showed that men in the *Low anxiety, high attitudes* profile were significantly less likely to be in a program with low math load. In women, those with the *Low math anxiety, high attitudes, medium general anxiety* were more likely to be in programs with high math load; those with the *Low anxiety, high attitudes* profile were more likely to be in programs with medium math load and less likely to be in programs with low math load; those with the *High anxiety, low attitudes* and *Low attitudes, medium rest* profiles were more likely to be in programs with low math load. However, these results should be carefully interpreted, given some cells exhibiting very small size (see Figure S1, https://osf.io/3ehqc). The interaction between profile and gender in explaining variance in the math load influence on study choice was statistically significant, but the low value of the Bayes factor points to evidence in favor of the null hypothesis (*F*(6, 823) = 2.19, *p* = 0.042, *BF*_*10*_ = 0.80, Figure S2, https://osf.io/3ehqc). Post hoc comparisons between men and women displaying the same profile were not statistically significant (all *p* > 0.05).

### 3.5 Profile Differences Beyond Mathematical Achievement

One might argue that the profile differences reported earlier could be confounded by mathematical achievement. Math attitudes and math anxiety are closely linked to mathematical skills, which in turn influence university program selection and the tendency to avoid mathematics. To account for this potential confound, we conducted additional exploratory (nonpreregistered) analyses to examine whether profile differences persist beyond differences in mathematical achievement.

To test the association between profile and math load, we ran two multinomial logistic models with math load as the dependent variable. First, we ran a model including math achievement as a covariate, then we additionally entered profile and we compared these two models with a likelihood‐ratio test for nested models, which resulted in statistically significant results (*χ* ^2^(12) = 41.64, *p* < 0.001), indicating a significant effect of profile.

Then, we performed an ANCOVA with the influence that math load had in the study program choice as dependent variable, profile as a factor and math achievement as covariate. Both the main effects of math achievement (*F*(5, 825) = 23.12, *p* < 0.001, *BF*_*10*_ > 100) and profile (*F*(6, 825) = 13.54, *p* < 0.001, *BF*_*10*_ > 100) were statistically significant.

Finally, we ran two binomial logistic regression models to test the association between profile and gender. In the first model, math achievement was entered as covariate and in the second model we added gender. A likelihood‐ratio test showed a significant difference between these two models (*χ* ^2^(6) = 70.12, *p* < 0.001), indicating a significant effect of gender.

Overall, these findings suggest that profile differences persist even after accounting for individual differences in mathematical achievement.

## 4 Discussion

This study investigated the specificity of math anxiety in relation to other types of anxiety (neuroticism, general anxiety, and test anxiety) and math attitudes (math self‐concept and self‐efficacy) in influencing university students’ program choice, mathematics avoidance, and related gender differences. Correlational analysis showed that math anxiety was positively associated with neuroticism, general, and test anxiety (0.33, 0.35, and 0.39, respectively) and negatively associated with math self‐concept and self‐efficacy (−0.64 and −0.50). Additionally, better arithmetic performance correlated with lower math anxiety (−0.25) and higher self‐concept and self‐efficacy (0.31 and 0.33). These results align with previous findings showing moderate to strong correlations of math anxiety with other types of anxiety (neuroticism: 0.25 and 0.41; general anxiety: 0.35 to 0.44; test anxiety: 0.52 to 0.64 [11, 16, 20, 21, 53]), with math attitudes (−0.68 to −0.29 [21, 54, 55]), and moderate correlations of arithmetic performance with math anxiety and attitudes (0.28 to 0.32 [21, 55, 56, 57]).

To better understand what drives university program choice, these factors should not be examined in isolation. One effective way to capture how multiple factors co‐occur within individuals is through LPA. Using this approach, different profiles of anxiety and attitudes toward mathematics were identified, each showing different associations with students' chosen university programs.

### 4.1 Profiles Description

Using LPA, we identified seven profiles of anxiety and math attitudes. As expected, a *High anxiety, low attitudes* profile, the opposite *Low anxiety, high attitudes* profile and a *Medium anxiety and attitudes* emerged. We additionally observed math‐specific profiles, such as the *High math anxiety, medium rest*, the *Low attitudes, medium rest*, and the *Low math anxiety, high attitudes, medium general anxiety* profiles. Finally, a profile characterized as *High general anxiety, medium rest* was also identified. These results align with previous studies showing the presence not only of extreme profiles, with high or low levels across anxiety types and one with the middle values on all variables, but also a differentiation in anxiety combinations, such as profiles with high general anxiety only or with high math anxiety only [21, 34]. Moreover, while high math anxiety often coexists with low math attitudes [32, 33], this is not always the case. Our results showed the presence of subgroups characterized either by high math anxiety and average math attitudes, or by low math attitudes and average math anxiety. Students with high math anxiety do not necessarily exhibit negative math attitudes, and vice versa students with negative math attitudes do not necessarily report high math anxiety, as previously shown among primary school and high school students [32, 33] and now also observed in university students.

The validity of these profiles was confirmed by differences in interests and performance. Participants displaying a profile characterized by positive attitudes toward mathematics reported more enjoyment of mathematics, lower preference for humanities, and better arithmetic performance compared to participants displaying the other profiles, especially those characterized by negative emotions and attitudes toward mathematics. According to these findings, engagement in mathematics seems to be explained more by positive math attitudes than by low math anxiety. Interestingly, omission rates in the arithmetic task were particularly low in the *Low anxiety, high attitudes* and particularly high in the *Low attitudes, medium rest* and the *High math anxiety, medium rest* profiles. This pattern suggests that negative attitudes and higher anxiety levels are associated with distinct approaches to engaging with arithmetic tasks.

### 4.2 Profile Differences

In line with our hypothesis, study program distribution and the influence math played in program choice were different across the profiles. Students in the profiles with positive attitudes and low (math) anxiety were more likely to be enrolled in programs with dense math content driven by their willingness to engage with mathematics. In contrast, students in the profiles with negative attitudes and/or high anxiety toward mathematics were more frequently found in programs with minimal mathematical content, reflecting their desire to avoid mathematics in their career. These findings align with previous research on the impact of math anxiety and attitudes on career choices [7, 8, 9, 28, 29]. Furthermore, they extend prior evidence by demonstrating that students’ specific patterns of math‐related attitudes and emotions is linked to the way the presence of mathematics influences study program selection. While primarily positive or negative attitudes toward math are decisive regarding the study program, high math anxiety additionally leads to additional avoidance of math when attitudes are rather neutral.

Moreover, the distribution of men and women across profiles differed. Women were more likely to belong to profiles with low attitudes, and less likely to the *Low anxiety, high attitudes* profile. This aligns with previous findings showing that women tend to report higher math anxiety and lower attitudes toward mathematics than men [12, 13, 27]. Additionally, profiles were differently associated with the type of study program across gender. Women, but not men, in the *Low math anxiety, high attitudes, medium general anxiety* profiles were more likely to choose a math‐intensive program, while women in the *Low anxiety, high attitudes* profile more often selected programs with moderate math content. This suggests that many women with positive attitudes toward mathematics still tend to choose fields where math plays a secondary role. One possible explanation is the influence of math‐gender stereotypes, that is, the false belief that mathematics is a male domain [58]: despite their positive attitudes, some women may have avoided highly math‐focused programs due to these societal perceptions, opting instead for fields perceived as “less masculine.” However, the evidence for a gender difference in the relationship between profiles and math avoidance was inconclusive. It is possible that attitudes and anxiety patterns outweigh the effect of gender. In other words, individual differences in math‐related emotions may be more relevant than gender in determining how mathematics influences program selection. These results should be interpreted with caution, though, as some groups had very small sample sizes. For instance, no women in the *Low Attitudes, Medium Rest* or *High Anxiety, Low Attitudes* profiles were enrolled in high math programs. While this aligns with our reasoning above, it also limits the statistical robustness of these findings.

Overall, when examining how profiles align with students' study program choices, their tendency to avoid math (as well as gender differences), a clear pattern emerges: profiles are primarily ordered by math attitudes rather than math anxiety. Only when attitudes are not particularly high or low, math anxiety seems to play a secondary role for profile differentiation. Importantly, these profile differences persist even when controlling for math achievement, ruling out the possibility that the observed effects are a consequence of mathematical performance [59].

### 4.3 Implications and Future Directions

The insights from this study have important theoretical and practical implications. On a theoretical level, focusing solely on the effects of math anxiety on mathematical performance or achievement may overlook the role of other individual characteristics and particularly math attitudes that may exert an even stronger influence, as suggested by our data. Research suggests that when students’ beliefs and perceptions of their mathematical abilities are considered, effects of math anxiety on achievement and enrollment intentions are mostly indirect [59]. It is essential to consider math attitudes in research on math anxiety to better understand their reciprocal influence and their respective contribution toward math‐related outcomes. These constructs follow interconnected developmental trajectories, influencing and being influenced by math achievement over time [60]. Math self‐concept differentiates from other academic self‐beliefs, such as language self‐concept, in the early years of primary school [61, 62, 63] and predicts future math outcomes, which in turn can reinforce individual beliefs [62, 64, 65]. Math attitudes and math anxiety also show reciprocal longitudinal influences, where more positive attitudes predict later lower math anxiety and vice versa [54, 64, 65]. However, developmental patterns of math attitudes and math anxiety may vary across individuals, with some students maintaining positive attitudes and low anxiety, while others experience a decrease in math attitudes and an increase in math anxiety, or show nonlinear patterns [66]. These differences suggest that the profiles identified in this study may also change over time [34]. Future research should investigate profiles of math anxiety and math attitudes in younger populations to track their development.

From an educational perspective, early and precise identification of such profiles can help target children at risk of underachievement or avoidance of mathematics more effectively than measuring math anxiety alone. Moreover, fostering positive attitudes toward mathematics is just as crucial as addressing math anxiety to ensure that students have equal access to all career paths. Improving self‐beliefs can boost math achievement by increasing efforts in mathematics [67] and persistence in math tasks [68]. It may also indirectly reduce math anxiety, as math self‐concept helps to better deal with negative feedback [68], which is a potential source of stress during math tasks [69]. Interventions aimed at improving math self‐concepts have already shown efficacy in enhancing math outcomes (for a review of interventions in middle school, see Ref. 70). These interventions, by improving self‐beliefs in math, can also reduce math anxiety, although it has yet to be empirically tested.

These findings have also important implications for policy makers. Reducing the gender gap in STEM has been a key goal of institutions worldwide [71]. However, this gap still persists even in countries with higher gender equality [4]. Our study suggests that this trend is supported not only by math anxiety but also by girls’ and women’ negative perceptions of their math abilities. Gender differences in math attitudes emerge during primary school and tend to remain stable in time [72]. Policies aimed at reducing the gender gap in STEM should consider programs targeting girls’ and women's math attitudes starting from the early years of schooling, as their improvement may counteract the pervasive effect of gender stereotypes [73].

### 4.4 Limitations

These results should be interpreted in light of some limitations. First, our measure of math avoidance in university program selection relies on retrospective self‐evaluation, which may be subject to consistency bias, that is, the tendency to interpret past behavior in a way that aligns with their current self‐image [74]. Additionally, our data do not allow for causal inference. While we interpreted profile differences as influencing study choice, we cannot rule out the possibility that the profiles are a consequence of being in a particular academic program. Profile membership is not necessarily stable, and shifts from one profile to another are possible and can be influenced by contextual factors [75]. Longitudinal research is needed to clarify these relationships.

Second, gender differences in self‐reported measures may be influenced by response bias, specifically, women's tendency to report their emotions, particularly negative emotions such as anxiety, more readily than men [76, 77]. This effect is further moderated by social desirability [78], which may lead to systematic differences in how men and women report their experiences. Moreover, the generalizability of our findings is limited by the educational system and the cultural context.

## 5 Conclusions

In conclusion, our findings demonstrate that math avoidance at university is not due to math anxiety only. Profiles combining different anxiety types with math attitudes are linked to math avoidance in young adults when selecting a university program. The presence of mathematics can influence program choices, potentially deterring individuals from pursuing their desired careers. The pattern of our profiles suggests that attitudes are the primary and key driver of the study program choice and math anxiety only comes into play as a secondary driver when math attitudes are neutral. Therefore, fostering positive attitudes toward math can also play a crucial role in enabling individuals to reach their full potential, allowing students to explore a wider range of careers without being constrained by external factors such as stereotypes.

## Author Contributions

**Maristella Lunardon**: conceptualization, formal analysis, visualization, writing – original draft preparation, writing – review and editing. **Christina Artemenko**: conceptualization, methodology, project administration, writing – review and editing. **Serena Rossi**: formal analysis, writing – review and editing. **Hans‐Christoph Nuerk**: supervision, writing – review and editing. **Krzysztof Cipora**: conceptualization, methodology, supervision, writing – review and editing.

## Conflicts of Interest

The authors declare no conflicts of interest.

## Data Availability

The data that support the findings of this study are openly available in Open Science Framework at https://osf.io/z5egb/.
